# Marchand on Peroxide of Hydrogen, Glycozone and Hydrozone

**Published:** 1895-06

**Authors:** 


MARCHAND ON PEROXIDE OF HYDROGEN, GLYCOZONE AND HYDROZONE.—The therapeutical applications of Peroxide of Hydrogen (medicinal), Glycozone and Hydrozone, by Charles Marchand, Chemist. Ninth Edition.

This book of 200 pages, which contains all information on the subject, with reprints of elaboeate articles by leading contributors to Medical Literature, will be mailed to doctors mentioning this publication,

Send full address to Charles Marchand, 28 Prince St., New York.

